# Unlocking the genetic control of early seedling resistance to wheat powdery mildew through microphenomics

**DOI:** 10.1002/ps.70312

**Published:** 2025-10-28

**Authors:** Amira M.I. Mourad, Hanaa M.S. Ibrahim, Stefanie Lück, Andreas Börner, Dimitar Douchkov

**Affiliations:** ^1^ Leibniz Institute of Plant Genetics and Crop Plant Research (IPK) Seeland Germany; ^2^ Department of Agronomy, Faculty of Agriculture Assiut University Assiut Egypt; ^3^ Department of Microbiology, Faculty of Agriculture Cairo University Giza Egypt

**Keywords:** single locus genome‐wide association study, multilocus genome‐wide association study, gene enrichment, selection, genetic distance, BluVision

## Abstract

**Background:**

Powdery mildew is one of the most devastating diseases affecting wheat‐growing areas worldwide. The most effective strategy for managing this disease is through the cultivation of resistant genotypes.

**Results:**

In this study, early resistance was assessed in a set of 197 spring wheat genotypes using a novel Microphenomics approach. Parameters measured using this technique were: the number of micro‐colonies 48 h post‐infection (N48), the median colony area 72 h post‐infection (M72), and the area under the disease progress curve (AUDPC). No significant correlations were found among these parameters. Single‐locus and multi‐locus GWAS revealed 57, 61, and 57 significant markers associated with N48, M72, and AUDPC, respectively. These markers were located within 29, 42, and 34 genes, respectively. Gene enrichment analysis uncovered 17 biological processes (BP), 13 cellular components, and one molecular function pathway. The 17 BP pathways formed a single network controlled by five gene models. Eight genotypes were identified as highly resistant based on all evaluation parameters.

**Conclusion:**

Microphenomics is a cutting‐edge technique that facilitates large‐scale quantification of the early, often critical stages of plant‐pathogen interactions, providing unprecedented insights into the infection process and enabling the discovery of new resistance mechanisms. SL and ML‐GWAS, along with gene enrichment analysis, provided a deeper understanding of the genetic control of WPM early resistance. Genotypes from Oman and the UK were particularly notable as good sources for improving early resistance to wheat powdery mildew due to their superior response and the high genetic distance between them and the German genotypes. © 2025 The Author(s). *Pest Management Science* published by John Wiley & Sons Ltd on behalf of Society of Chemical Industry.

## Graphical abstract

Microphenomics is a new technique for detecting the very early resistance of powdery mildew in wheat seedlings, after 24, 48, and 72 h from infection. A number of 29, 42, and 34 genes models were identified to harbor significant SNP markers associated with the early resistance to WPM. The gene enrichment analysis of these gene models confirms their association with the resistance. Genotypes from Oman and the UK were notable as good sources for improving early resistance to WPM.

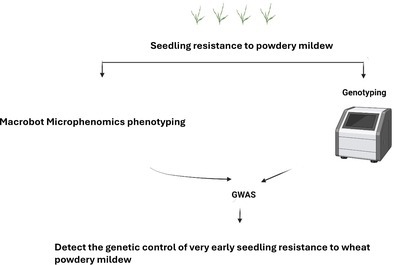

## 1 INTRODUCTION

Wheat (*Triticum aestivum* L.) is among the most vital cereal crops globally, serving as a staple food for approximately 35% of the world's population.^1^, ^2^ A major factor affecting wheat yield globally is diseases, particularly foliar fungal diseases, due to their ability to spread rapidly among plants. Once infection occurs, plants become weakened and more susceptible to other stresses.^3^

Among the various foliar diseases affecting wheat, powdery mildew (WPM), caused by *Blumeria graminis* f. *sp. tritici* (*Bgt*), is a major concern that has received comparatively less attention from wheat breeders than rust diseases such as yellow rust (*Puccinia striiformis* f. *sp. Tritici*), leaf rust (*Puccinia triticina*), and stem rust (*Puccinia graminis* f. *sp. Tritici*).^4^ Under favorable conditions, WPM has been reported to reduce wheat yields by up to 62%.^5^, ^6^, ^7^ This yield loss is anticipated to rise as climate change promotes the emergence of new and more aggressive pathogen races.^8^

*Bgt* is an obligate biotrophic ascomycete capable of both sexual and asexual reproduction. This enables it to spread rapidly and adapt genetically.^9^, ^10^ Therefore, developing effective strategies to improve wheat resistance against *Bgt* is urgently needed. Various methods have been employed to control this serious disease, including the application of fungicides, biological control,^11^, ^12^, ^13^ and breeding resistant genotypes. Among these, breeding resistant genotypes is considered the most effective strategy, given the harmful effects of fungicides on human health and the environment, as well as the limited knowledge available on biological control.^14^

Similar to other fungal diseases in wheat, resistance to WPM is typically categorized into seedling resistance, also known as all‐stage resistance (ASR), and adult‐plant resistance (APR). Unlike APR, ASR is a type of race‐specific resistance controlled by single genes with single effects.^15^, ^16^ Consequently, combining multiple resistance genes within a single genotype can confer broad‐spectrum resistance. To date, 68 wheat genes conferring seedling resistance to powdery mildew have been discovered.^17^ However, some of these genes have been found to exert deleterious effects on wheat plants.^18^

Significant efforts have been made to better understand the genetic control of wheat ASR against *Bgt*. However, relatively few studies have focused on the early interactions between *Bgt* and wheat.^19^, ^20^ One way to evaluate early resistance of WPM is the BluVision Micro platform, developed by the Leibniz Institute of Plant Genetics and Crop Plant Research (IPK), Germany. This technique provides valuable information on plant‐microbe interactions at the very onset of disease development using a microscopy slide scanner and machine learning based analysis to quantify the *Bgt* micro‐colony number and individual colony area at 48 and 72 h after infection.^19^, ^20^, ^21^ Unlike conventional visual scoring, the BluVision Micro platform provides wheat breeders and researchers with essential insights into early‐stage resistance to this significant disease. It also helps reveal novel mechanisms that may not be detectable through final infection outcomes alone, thereby facilitating the development of wheat genotypes with improved resistance to WPM from the earliest stages of growth. The use of a fully controlled environment combined with unbiased automated phenotyping further enhances the precision and reliability of evaluations.

Previous studies have explored the genetic control of WPM seedling resistance in winter wheat through precise quantification of disease severity, defined as the percentage of leaf area affected by the pathogen. These investigations utilized the BluVision Macro (Macrobot) module within the same phenotyping facility at IPK Gatersleben.^19^, ^20^ In the present study, a diverse collection of spring wheat germplasm obtained from 22 different countries was used to investigate genomic regions controlling the earliest resistance responses and underlying mechanisms. Diverse wheat germplasm is ideal for detecting new sources of desirable traits through association mapping (AM).^22^, ^23^, ^24^ AM has been extensively used in wheat breeding to enhance resistance to various biotic and abiotic stress traits.^25^, ^26^, ^27^, ^28^, ^29^

With advancements in sequencing methods, the entire wheat genome has been approximately covered with molecular markers using Genotyping‐by‐Sequencing (GBS) and the iSelect SNP array.^11^, ^30^, ^31^, ^32^, ^33^, ^34^, ^35^ Moreover, gene models containing markers significantly linked to specific traits can be easily identified through online databases such as *Ensemble Plants*.^36^ The functions of these gene models and their roles in controlling specific traits, as well as their expression levels, can be further explored using databases such as *WheatExp*,^37^ *Knetminer*,^38^ and *ShinyGo*.^39^ Combining data from these valuable resources with AM results will accelerate wheat breeding efforts to improve targeted traits.

The objectives of this study are to: (1) investigate the genetic control of early resistance to WPM using a highly diverse spring wheat germplasm and a novel phenotyping approach, (2) identify marker‐trait associations (MTAs) related to early resistance against the highly aggressive *Bgt* European isolate (FAL 92315), and (3) select the most resistant spring wheat genotypes that can be used to enhance early resistance to WPM.

## 2 MATERIALS AND METHODS

### 2.1 Plant materials

A total of 197 spring wheat genotypes were used in the current study. These genotypes were gathered from 22 countries around the world and contain breeding lines as well as old and new cultivars (Table S1). The majority of the tested genotypes were Egyptian (35 genotypes), while the remaining genotypes belonged to Morocco (15), Iran (14), Saudi Arabia (14), Australia (11), Germany (11), Oman (11), Afghanistan (9), Algeria (9), USA. (9), Kazakhstan (8), UK (7), Canada (6), Ethiopia (5), Kenya (5), Sudan (5), Greece (4), Tunisia (4), Italy (2), Syria (2), and Pakistan (one). Seeds of non‐Egyptian genotypes were acquired from USDA‐ARS in Aberdeen, ID, United States, while seeds for Egyptian genotypes were sourced from different Egyptian governorates.

### 2.2 Phenotyping and statistical analysis of early seedling resistance to powdery mildew

The phenotypic data for early seedling resistance were measured using the BluVision Micro facility developed at the IPK, Gatersleben, Germany. A complete description of the plant growing and sampling method is provided by Hinterberger *et al*. (2022).^40^ Briefly, 10 seeds from each tested genotype were planted in trays with 4 × 6 slots and placed in a greenhouse under controlled climate conditions (approx. 20°C day temperature and 17°C at night). After 15 days, the second leaves were collected from seedlings, with a total of eight leaves for each tested genotype. A 2‐cm segment was cut from the middle part of each leaf and placed onto 4‐well microtiter plates containing agar (1% water agar supplemented with 20 mg L^−1^ benzimidazole as a leaf senescence inhibitor). Two repetitions were performed for each tested genotype.

Using an inoculation tower, prepared leaf segments on plates were inoculated with the *Bgt* isolate FAL 92315. Fresh spores, collected from heavily powdery mildew‐infected wheat plants, were introduced into the tower using compressed air, generating strong turbulence to ensure even spore distribution. During inoculation, the base of the tower—holding the open plates—rotated to promote uniform spore deposition across the leaf surfaces. To monitor spore density, a microscope slide was placed on the base of the tower alongside the plates, with spore densities measured at 5–10 spores/mm^2^.^21^ Furthermore, a susceptible German check ‘Kanzler, TRI 13582’ was included in each inoculation as a running control.^40^, ^41^, ^42^ After inoculation, the plates were sealed with lids and transferred to an environment‐controlled incubation chamber set to 20°C, 60% relative humidity, and 16 h light (μE m^−2^ s^−1^). After incubation, the eight evaluated leaves from each genotype were separated into two groups. In the first one, the infection process was stopped 48 h after infection (hai) by dropping the leaves in a clearing solution 7 parts (v/v) 96% ethanol, and 1 part (v/v) acetic acid). The second group stopped at 72 hai. Both sample types were stained with trichloroacetic acid (7.5% (w/v), Coomassie staining solution (0.3% Coomassie R250, and 50% (v/v) Methanol) for 5 min, followed by multiple washes with water. The prepared samples were placed on microscope slides with 50% glycerol to prevent the leaves from drying out during image capture. Multimodal images of each group were automatically acquired with a Zeiss AxioScan.Z1 high‐performance slide scanner and analyzed using the in‐house developed BluVision Micro pipeline.

From these images, key parameters were measured: 1) the number of micro‐colonies observed 48 h post‐infection (N48), serving as a marker for penetration resistance and very early defense responses; 2) the median projected area of the hyphae of each colony after 48 h (M48), measured in pixel, reflecting pathogen growth 3) the median projected area of the hyphae of each colony after 72 h (M72), measured in pixel, reflecting pathogen growth; and 4) the area under the disease progress curve (AUDPC) that was calculated using the following equation

$$\text{AUDPC}=\sum_{i=1}^{n-1}\frac{Y_{i}+Y_{i+1}}{2}\;x\left(t_{i+1}–t_{i}\right)$$

where *y*_i_ is median colony at the *i*th observation, *t*_i_ is time in days at the *i*th observation, and *n* is the total number of observations.^43^

### 2.3 Estimation of statistical components

Variance components of the analyzed phenotypic parameters were estimated using a linear mixed model with the lme4 package.^44^ The following mixed model was used for each parameter

$$y_{\mathrm{ijk}}=\mu +G_{i}+R_{j}+E_{k\left(j\right)+}\epsilon_{\mathrm{ijk}}$$

where: *y*_*ijky*_: The observed value for the *i*‐th genotype, *j*‐th replication, and *k*‐th experiment nested within replication. *μ*: The overall mean (fixed factor). *G*_*i*_: The effect of the *i*‐th genotype (random). *R*_*j*_: The effect of the *j*‐th replication (random). *E*_*k(j)*_: The effect of the *k*‐th experiment nested within the *j*‐th replication (random). *ϵ*_*ijk*_: The residual error term (random).

The Best Linear Unbiased Estimations (BLUEs) of each genotype were computed using the same R package. Broad‐sense heritability (*H* ^2^) was estimated using the following equation

$$H^{2}=\frac{\delta_{G}^{2}}{\delta_{G}^{2}+\frac{\delta_{e}^{2}}{R}}$$

where $\delta_{G}^{2}$ is the genotypic variation, $\delta_{e}^{2}$ is the residual variance, and *R* is the average number of replications per genotype.

### 2.4 Association mapping of EWPM resistance

The tested wheat panel was genotyped using two different sequencing methods: genotyping‐by‐sequencing (GBS) and the 25K Infinium iSelect SNP array (25K‐SNPs). The GBS data were available for 103 genotypes, while 25K‐SNPs were available for the whole tested panel. Both markers were generated and filtered as described in Mourad *et al*. (2020, 2022, 2023),^15^, ^45^, ^46^ Esmail *et al*. (2023),^47^ and Mourad *et al*. (2024).^24^, ^48^ After filtration, a total number of 21 093 and 36 720 SNPs were available for 25K‐SNPs and GBS‐SNPs, respectively.

To better understand the genetic control of the early response of wheat seedlings to WPM, Best Linear Unbiased Estimates (BLUEs) for all studied parameters were used to perform Genome‐Wide Association Studies (GWAS). GWAS was conducted using two methods: single‐locus GWAS (SL‐GWAS) and multi‐locus GWAS (ML‐GWAS).

SL‐GWAS was performed using rMVP package^49^ with three different models: the Generalized Linear Model (GLM), the Mixed Linear Model (MLM), and the Fixed and Random Model Circulating Probability Unification (FarmCPU), incorporating kinship and/or PCA as covariates. The best model was selected based on the QQ‐plot, which shows the deviation of the observed −log_10_ (*P*‐value) distribution from the expected values. Significant markers were defined as those with *P*‐values ≤0.001 (−log_10_ ≥ 3.00).

ML‐GWAS was performed using six different models: pLARmEB, ISIS EM‐BLASSO, mrMLM, FASTmrMLM, FASTmrEMMA, and pkWmEB implemented in the mrMLM v.4.0.2 R package.^50^ The significant markers were those with −log_10_ (*P*‐value≥3.00).

For both GWAS methods, the target allele at each significant marker was the one that reduced all the parameters studied. TASSEL software was employed to calculate the phenotypic variation explained by the significant markers (R^2^) identified through SL‐GWAS.^51^

### 2.5 Gene models harboring the identified significant markers, their functional annotations, and gene enrichment

Gene models containing the significant markers were identified using the *Ensemble Plants* ^36^ database, based on the base pair positions of the markers and the presence of corresponding gene models at those positions. The functional annotation of these gene models was obtained using the International Wheat Genome Sequencing Consortium (IWGSC) v.2.0. Gene enrichment analysis of the identified gene models was performed using the *ShinyGo* 0.76 database^39^ and focused on pathways related to biological process, molecular function, and cellular components. A false discovery rate (FDR) cutoff of *P* <0.05 was applied during the gene enrichment analysis to identify the most significant pathways.

## 3 RESULTS

### 3.1 Genetic variation of powdery mildew early resistance in wheat seedlings

The susceptible check showed a high level of infection in both repetitions (Table S2). The average of ‘Kanzler’ in the first repetition was 93.54, 4831.74, 25 481.76, and 597508.89 for N48, M48, M72, and AUDPC, respectively. In the second replication, the corresponding values were 138.72, 13,473.01, 30,945.31, and 1,207,659.62, respectively.

**Figure 1:**
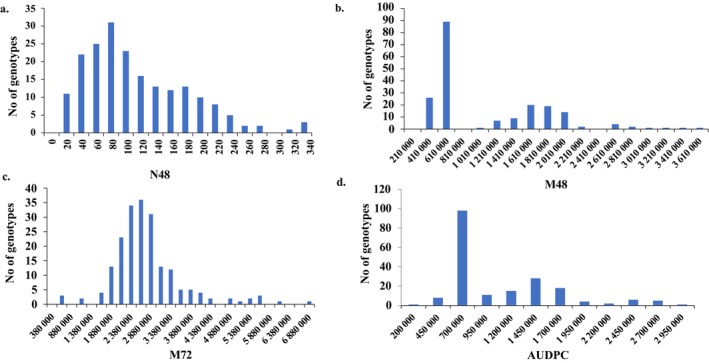
Distribution of number of colonies after 48 h from infection (N48) (a), median area of colonies after 48 h of infection (M48) (b), median area of colonies after 72 h of infection (M72) (c), and area under disease progress curve (AUDPC) (d) in the tested 197‐spring wheat genotypes.

Significant variation in the early response to *Bgt* was observed among the tested genotypes based on the studied parameters. The normalized number of colonies after 48 h (N48) ranged from 3.16 to 325.02 colonies, with an average of 105.20 colonies (Fig. 1a). The median area of hyphae in each colony after 48 h (M48) ranged from 218 316 to 3 606 163.20 pixels, with an average of 980 396.12 pixels (Fig. 1b). The median area after 72 h (M72) ranged from 384 600 to 6 842 016 pixels, with an average of 2 666 471.36 pixels (Fig. 1c). The AUDPC ranged from 195 265.50 to 2 735 586, with an average of 965 414.96 (Fig. 1d). High variance was observed across the tested genotypes for N48, M72, and AUDPC, while no significant variance was found for M48 (Table 1).

**Table 1:** Variance component of the raw dataset for number of colonies after 48 h (N48), median colony area after 48 h (M48), median colony area after 72 h (M72), and area under disease progress curve (AUDPC)

	N48		M48		M72		AUDPC	
	Variance	Std. Dev.	Variance	Std. Dev.	Variance	Std. Dev.	Variance	Std. Dev.
Genotype	1315	36.26	0	0	295E+07	5433.90	1.13E+09	33 608
Replication	0	0	0	0	0.078	6307.88	0	0
Exp: Rep	02003	44.75	7394	85.99	2.98E+07	0.28	3.40E+11	582 843
Residual	3255	57.05	3600	60	4.48E+07	6689.71	1.54E+11	392 756
Heritability (*H* ^2^)	0.57		‐		0.60		0.66	

**Figure 2:**
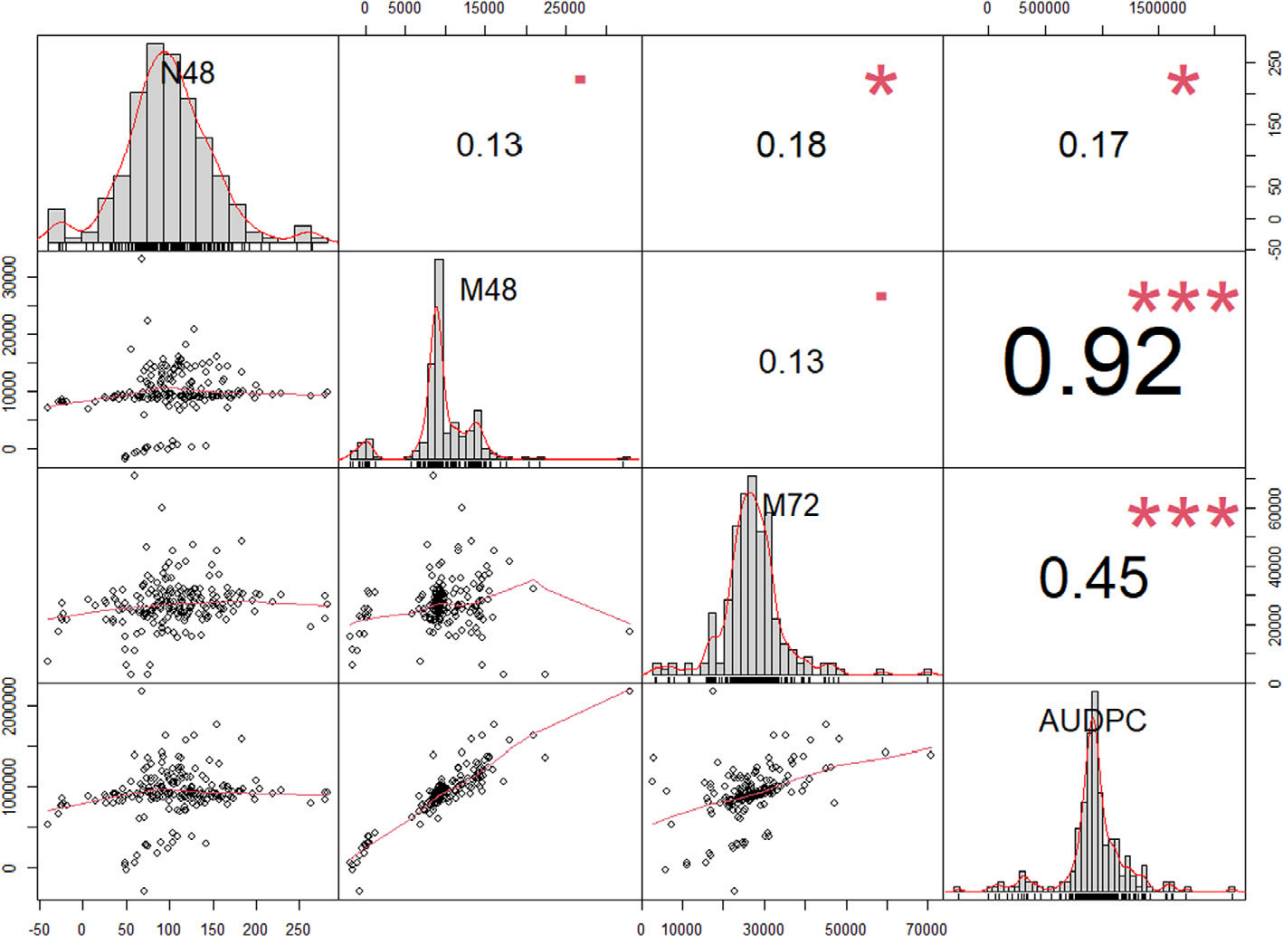
Phenotypic correlations of early seedling resistance to powdery mildew in 197 spring wheat genotypes.

A significant positive correlation was observed between N48 and both M72 and AUDPC. While no significant correlation was observed between N48 and M48. Furthermore, M48 showed a significant positive correlation with AUDPC. No significant correlation was detected between M48 and both N48 and M72 (Fig. 2).

### 3.2 Association mapping of powdery mildew early seedling resistance

The tested genotypes did not show any variation for M48; therefore, this parameter was excluded from the GWAS. For the remaining three parameters, two different GWAS methods were used: single‐locus GWAS (SL‐GWAS) and multi‐locus GWAS (ML‐GWAS). SL‐GWAS was performed using different models for each parameter with both GBS‐SNPs and 25K‐SNPs. Based on the QQ‐plot of each studied model, the FarmCPU + PCA + Kin model proved to be the most effective for all studied parameters, using both types of markers, except for M72 and AUDPC with GBS‐SNPs, where the optimal models were MLM + PCA + Kin and FarmCPU+Kin, respectively (Fig. S1). These GWAS models detected a total of 46, 38, and 29 significant markers associated with N48, M72, and AUDPC, respectively (Table 2, Table S3, and Fig. S2). The phenotypic variation explained by significant markers (*R* ^2^) associated with N48 ranged from 1.03% to 42.25%. The 38 markers significantly associated with M72 accounted for 1.20% to 38.96% of the phenotypic variation and were located within 31 gene models. Meanwhile, the 29 markers significantly associated with AUDPC explained between 1.01% to 27.97% of the phenotypic variation and were located within 17 distinct gene models.

**Table 2:** Summary of significant markers associated with number of colonies after 48 h (N48), median colony area after 48 h (M48), median colony area after 72 h, and area under disease progress curve (AUDPC) based on single‐locus genome‐wide association study (SL‐GWAS) using i‐select 25 K‐SNP array (25 K‐SNPs) and genotyping‐by‐sequencing (GBS) marker data sets

Experiment	Marker set	No. of sig. markers	*R* ^2^ (%)	*P*‐value	Allele effects	No. of gene models
N48	25 K‐SNPs	25	1.03–10.11	8.13E‐06–0.001	(−32.84) to (−14.10)	17
	GBS‐SNPs	21	1.17–42.25	4.00E‐06–0.001	(−47.15) to (−21.66)	3
	Total	46	1.03–42.25	4.00E‐06–0.001	(−47.15) to (14.10)	20
M72	25 K‐SNPs	31	1.20–11.70	6.59E‐05–0.001	(−4443) to (−2121)	26
	GBS‐SNPs	7	2.01–38.96	0.0002–0.001	(−8283) to (−3042)	5
	Total	38	1.20–38.96	6.59E‐05–0.001	(−8283) to (−2121)	31
AUDPC	25 K‐SNPs	13	1.01–27.97	5.58E‐08–0.001	(−214 141) to (−47 806)	12
	GBS‐SNPs	16	1.08–23.79	1.08E‐08–0.001	(−95 707) to (−42 708)	5
	Total	29	1.01–27.97	5.58E‐08–0.001	(−214 141) to (−42 708)	17

ML‐GWAS identified a total of 17, 28, and 30 markers that were significantly associated with N48, M72, and AUDPC, respectively (Table 3, Fig. S3, Table S4, and Fig. S4). The 17 significant markers associated with N48 accounted for 2.40% to 18.78% of the phenotypic variation and were located within 12 distinct gene models. The significant markers associated with M72 explained 1.89% to 21.92% of the phenotypic variation and were found within 14 different gene models. The 30 markers associated with AUDPC explained phenotypic variation ranging from 0.44% to 18.78% and were located within 18 distinct gene models.

**Table 3:** Summary of significant markers associated with the number of colonies after 48 h (N48), median colony area after 48 h (M48), median colony area after 72 h, and area under disease progress curve (AUDPC) based on multi‐locus genome‐wide association study (ML‐GWAS) using iselect 25K‐SNP array (25 K‐SNPs) and genotyping‐by‐sequencing (GBS) marker data sets

Experiment	Marker set	No. of sig. markers	*R* ^2^	‐log_10_(*P*‐value)	Allele effects	No. of gene models
N48	25 K‐SNPs	13	2.40–10.74	3.72–5.96	(−28.02) to (−9.45)	11
	GBS‐SNPs	4	4.52–18.78	3.74–4.94	(−45.60) to (−13.49)	1
	Total	17	2.40–18.78	3.72–5.96	(−45.60) to (−9.45)	12
M72	25 K‐SNPs	8	2.65–7.54	3.82–5.67	(−4261) to (−1384)	7
	GBS‐SNPs	20	1.89–21.92	3.92–12.77	(−4456) to (−1109)	7
	Total	28	1.89–21.92	3.82–12.77	(−4456) to (−1109)	14
AUDPC	25 K‐SNPs	20	0.44–16.37	3.27–6.65	(−487 313) to (−34 944)	15
	GBS‐SNPs	10	4.17–18.78	3.79–6.07	(−298 349) to (−86 424)	3
	Total	30	0.44–18.78	3.27–6.65	(−487 313) to (−34 944)	18

**Figure 3:**
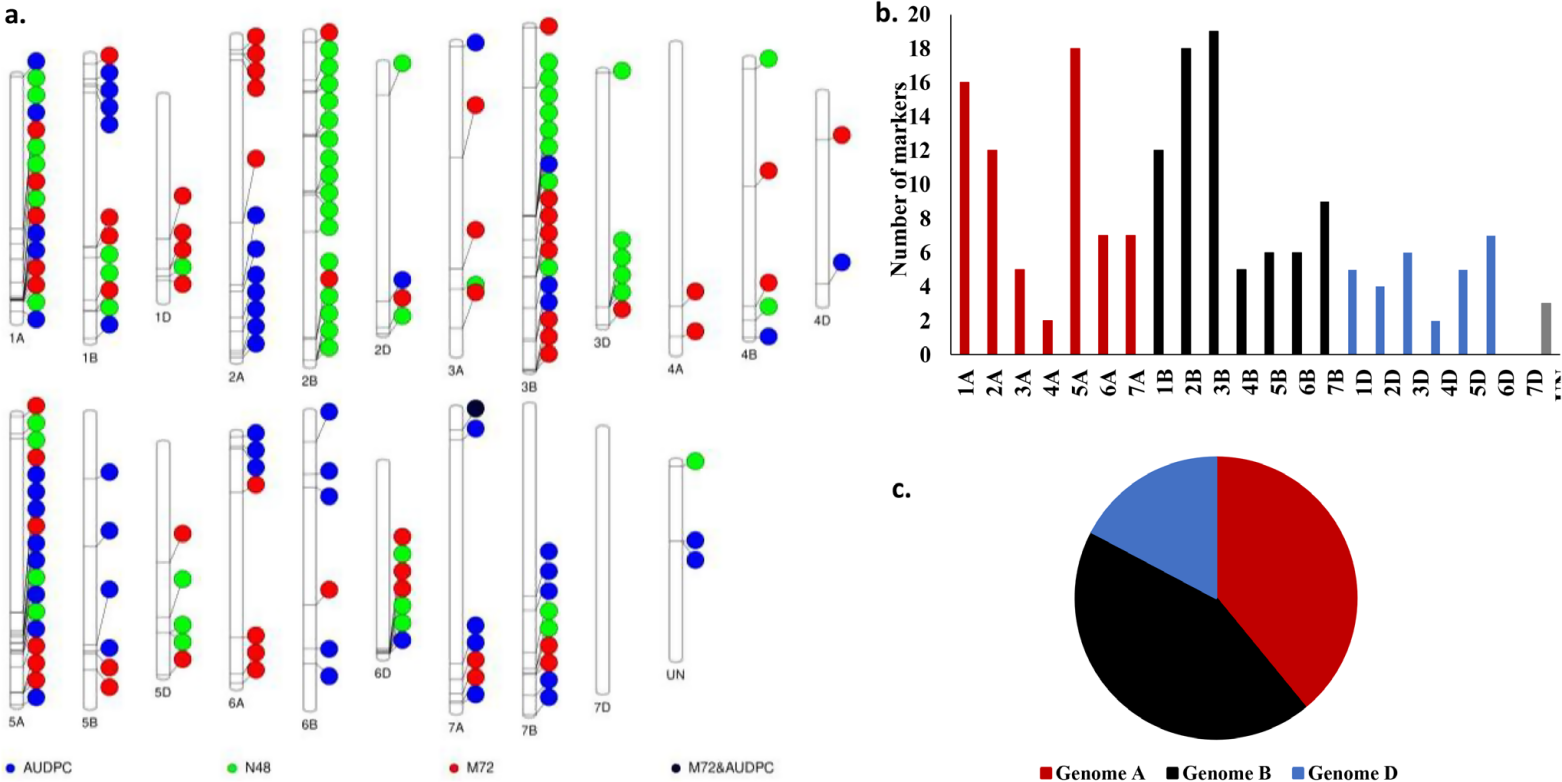
Number of significant markers associated with the number of colonies after 48 h from infection (N48), median area of colonies after 72 h of infection (M72), and area under disease progress curve (AUDPC). (a) chromosomal position of significant markers associated with each trait, (b) distribution of the significant markers on the 21‐wheat chromosomes, and (c) distribution of the significant markers on each wheat genome.

By combining the results of SL‐GWAS and ML‐GWAS, a total of 57, 61, and 57 significant markers were identified as being strongly associated with N48, M72, and AUDPC, respectively. Out of these markers, six, five, and two were commonly associated with N48, M72, and AUDPC based on the two GWAS methods (Fig. S5). The significant markers associated with N48 were distributed across 14 different chromosomes, while those associated with M72 and AUDPC were distributed across 20 and 16 chromosomes, respectively (Fig. 3a). Among the identified significant markers, only one marker (S7A_51491042) was commonly associated with both M72 and AUDPC, and it was located on the 7A chromosome. This marker explained 9.09% and 19.26% of the phenotypic variation of M72 and AUDPC, respectively (Tables S2 and S3). In total, all 21 wheat chromosomes carried significant markers associated with at least one disease parameter, except for the 7D chromosome (Fig. 3b). The greatest number of significant markers was observed on chromosome 3B (19 markers), followed by chromosomes 5A and 2B, each carrying 18 markers. The fewest significant markers were found on chromosomes 4A and 4D, each containing two markers. The B genome had the highest proportion of significant markers (44%), followed by the A genome, which carried 39% (Fig. 3c), while the D genome carried the lowest percentage (17%).

### 3.3 Functional annotation and gene enrichment of gene models controlling early wheat powdery mildew resistance

**Figure 4:**
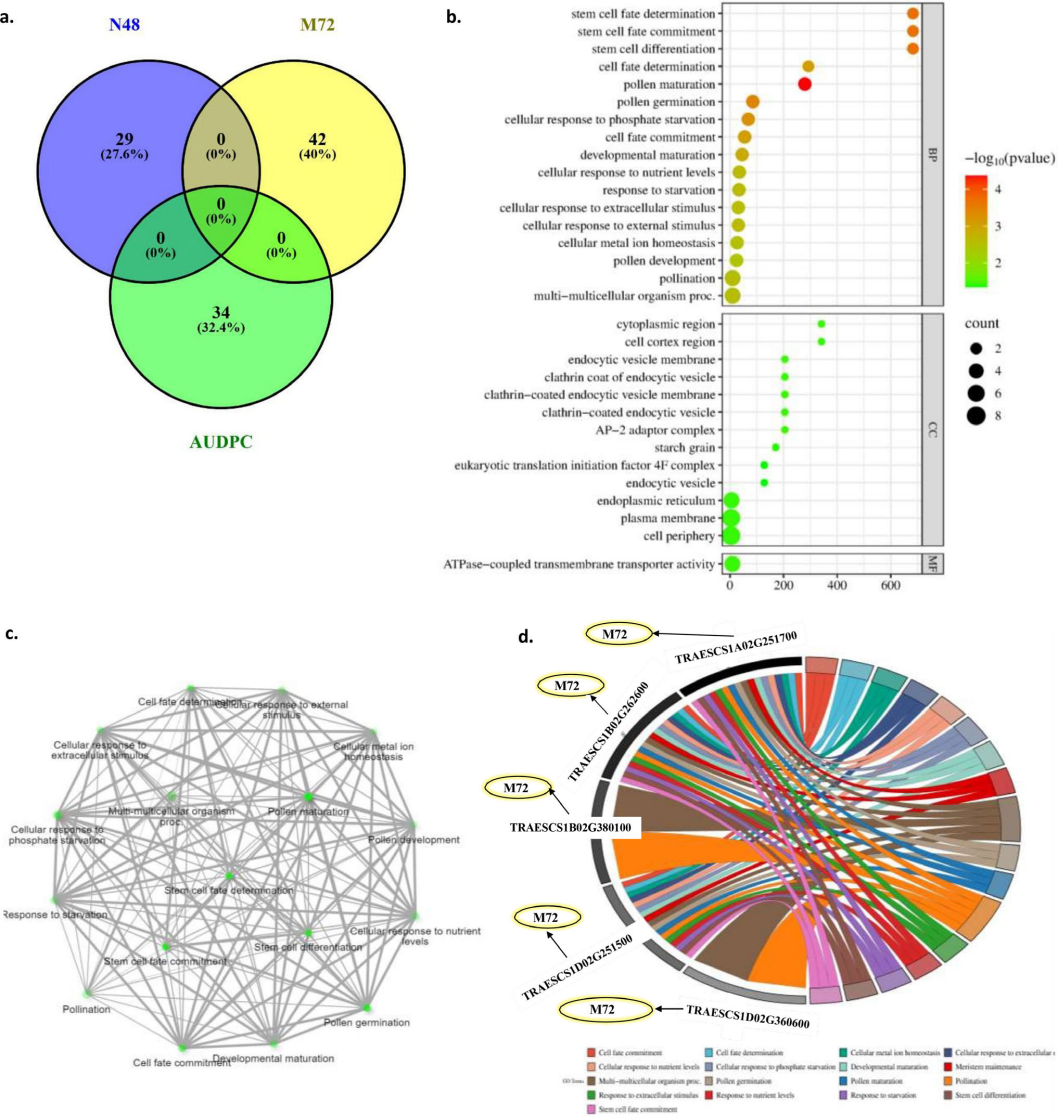
(a) Number of gene models harboring markers significantly associated with the early resistance of powdery mildew. (b) Gene enrichment analysis of the identified gene models controlling the resistance based on biological process, cellular components, and molecular function pathways, (c) Gene network of the identified gene models based on biological process pathways, and (d) genes controlling each pathway.

Both SL‐GWAS and ML‐GWAS methods identified 29, 42, and 34 genes containing significant markers associated with N48, M72, and AUDPC, respectively (Fig. 4a). The functional annotations of these gene models are provided in Tables S3 and S4. Most of the identified genes were functionally annotated as being involved in disease resistance. For example, ‘*TraesCS2D02G466000’* encodes an NBS‐LRR disease resistance protein‐like protein, ‘*TraesCS5A02G052400*’ is annotated as antifungal, and ‘*TraesCS1B02G260400*’ encodes a 1,4‐beta‐D‐glucanase enzyme.

To gain a deeper understanding of the genetic control of early WPM resistance, enrichment analysis of the identified gene models was conducted. A 1% FDR cutoff was applied to highlight the most significant genes. A total of 17 highly significant biological processes (BP), 13 cellular components (CC), and one molecular function (MF) were identified (Fig. 4b). The 17 BP pathways were found to function together within a single network, regulated by five distinct gene models containing markers significantly associated with M72 (Fig. 4c,d). Interestingly, most of these pathways are associated with stem cell differentiation and pollen maturation. However, some pathways are associated with other functions important for disease resistance, such as cellular metal ion homeostasis. On the CC level, the 13 pathways were found to control important functions in the cell membrane, such as clathrin‐coated endocytic vesicles and starch gain (Table S5).

### 3.4 Selection of the most resistant and superior genotypes

**Figure 5:**
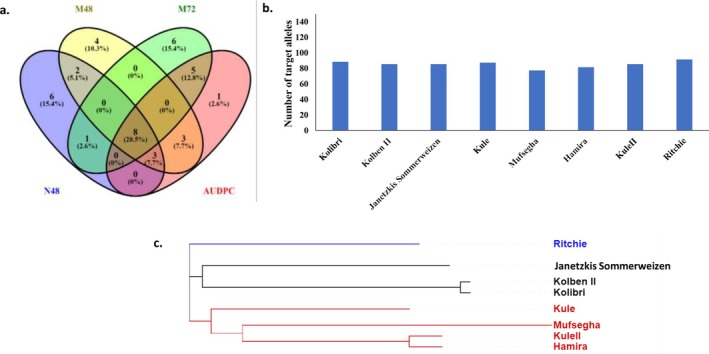
Selection of the best genotypes based on the very early resistance traits (a), number of target alleles in the eight selected genotypes (b), and the genetic distance between each pair of them (c).

To identify the best resistant genotypes, all the 197‐evaluated genotypes were sorted based on their response from lowest to highest, and the lowest 20 genotypes based on each parameter were selected. Eight genotypes were found to be common among the 20 selected genotypes based on each parameter (Fig. 5a). These genotypes came from three different countries: four from Oman, three from Germany, and one from the United Kingdom (Table 4). Notably, the UK genotype ‘Ritchie’ showed the lowest values for all parameters, followed by the two German genotypes, ‘Kolben II’ and ‘Kolibri’ based on resistance parameters after 48 h. Meanwhile, the Omani genotype ‘Mufsegha’ outperformed the German genotypes based on M72 and AUDPC.

**Table 4:** List of the best eight genotypes selected based on the studied resistant parameters (N48, M48, M72, and AUDPC)

Genotype	IP code	Country
Kolibri	PI_313101	Germany
Kolben II	PI_321700	Germany
Janetzkis Sommerweizen	PI_191600	Germany
Kule	PI_532249	Oman
Mufsegha	PI_532255	Oman
Hamira	PI_532258	Oman
Kule II	PI_532259	Oman
Ritchie	PI_279454	UK

To genetically assess the superior resistance in the selected genotypes, the number of significant markers associated with the different parameters studied was detected in each selected genotype (Fig. 5). This number ranged from 77 in the Omani genotype ‘Mufsegha’ to 91 in the UK genotype ‘Ritchie’. Notably, the UK resistant genotype carried 91 markers out of the 189 significant markers associated with N48, M72, and AUDPC. The two selected German genotypes had intermediate numbers of the significant markers, with 88 and 85 markers for ‘Kolibri’ and ‘Kolben II,’ respectively.

To validate the potential for improving wheat powdery mildew resistance using the selected genotypes, the genetic distance between each pair of genotypes was calculated. The eight‐selected genotypes were grouped into three distinct subpopulations based on their country of origin (Fig. 5c). Moreover, a high genetic distance was observed between each pair of tested genotypes, ranging from 0.014 between the two German genotypes ‘Kolibri’ and ‘Kolben II’ to 0.468 between the German genotype ‘Kolibri’ and the Omani genotype ‘Mufsegha’ (Table S6). A substantial genetic distance was also observed between the UK genotype ‘Ritchie’ and the two German genotypes, ‘Kolibri’ and ‘Kolben II,’ with a value of 0.376. The distance between ‘Ritchie’ and the Omani genotype was very high, with a value of 0.458.

## 4 DISCUSSION

A novel Microphenomics approach, which provides an accurate way to evaluate the early reaction between wheat seedlings and *Bgt* was used in the recent study. Furthermore, we used a highly diverse spring wheat panel that was reported to exhibit significant diversity in disease resistance. This study underscores the potential of the Microphenomics approach to provide a new perspective on disease resistance, enabling fine dissection of the infection process and uncovering novel resistance mechanisms critical for combating wheat powdery mildew. The susceptible check showed a high level of infection, confirming the success of the artificial inoculation and the reliability of the obtained data.

### 4.1 Genetic variation of early resistance of WPM

The evaluated wheat panel showed variances for all four parameters studied except M48. Furthermore, high broad‐sense heritability values were observed for these parameters, indicating that the observed variance is mainly due to genetic variation. Therefore, selecting genotypes exhibiting high levels of early resistance to WPM is possible using the current panel. High levels of broad‐sense heritability for WPM seedling resistance have been reported in previous studies.^15^, ^40^, ^52^

Notably, none of the tested genotypes had zero colonies on their leaves, validating the effectiveness of the artificial inoculation. However, a few genotypes (12 genotypes) showed a low number of colonies, with fewer than 20 colonies. The highest number of colonies was 325 per 2 cm, representing the high level of susceptibility in the studied panel and the potential need to select highly resistant genotypes. Interestingly, a low significant correlation was found between the number of colonies and their area, as well as between N48 and AUDPC, confirming that different resistance mechanisms are underlying these phenotypes. Furthermore, some genotypes with a low number of colonies had wider colonies that developed quickly, confirming different infection development. Therefore, we can conclude that all the studied parameters should be considered when selecting resistant genotypes.

### 4.2 Association mapping of EWPM

This recent study can be considered as one of the few that have focused on the genetic control of EWPM resistance.^40^ Generally, minimal consideration has been given to the genetic control of WPM during both seedling and adult growth stages, unlike other wheat diseases.^51^ A highly diverse panel was used and genotyped using two different marker sets covering different parts of the wheat genome, strengthening the reliability of the GWAS results.^44^, ^45^, ^52^ To conduct a comprehensive analysis of the genetic control of early WPM resistance, GWAS was performed using two distinct methods (SL‐GWAS and ML‐GWAS), which differ in their accuracy in identifying marker‐trait associations (MTAs) related to the trait under study.^53^, ^54^, ^55^, ^56^ Out of the 113 and 75 significant markers identified using SL‐GWAS and ML‐GWAS, respectively, only 14 markers were common between the two GWAS methods (Tables S3 and S4). Thus, combining both methods in the analysis provides a deeper understanding of the genetic control of EWPM resistance. Moreover, each GWAS method was run using different models that are effective in mitigating the effects of population structure and avoiding false associations.^57^, ^58^

A large number of significant markers were found to be associated with each early resistance parameter (Tables 2 and 3). These markers were located on different chromosomes throughout the wheat genome except 7D (Fig. 3), suggesting that the early resistance of WPM is a complex trait and influenced by a wide genetic base. A similar wide genetic system was previously reported to control WPM resistance^40^, ^59^, ^60^, ^61^. The identified markers were situated within 106 different gene models and were functionally characterized as contributing to disease resistance in wheat (Tables S3 and S4). For example, some gene models were identified as regulating the synthesis of proteins and enzymes that are important in enhancing plant defense, such as NBS‐LRR disease resistance protein, 1,4‐beta‐D‐glucanase, F‐box family protein, and zinc CCCH domain protein.^62^, ^63^, ^64^, ^65^, ^66^ Some genes were not directly associated with disease resistance, such as heat shock transcription factor controlled by *TraesCS2A02G089300*, glycerophosphodiester phosphodiesterase controlled by *TraesCS1A02G353200*, and receptor‐like protein kinase involved in salt stress response/antifungal controlled by *TraesCS2B02G241700* genes. However, these functions were also reported to control disease resistance in plants.^67^, ^68^, ^69^ Furthermore, *TraesCS4B02G307000* gene was reported previously to have a minor role in controlling WPM seedling resistance under Egyptian conditions.^15^ A previous study was carried out on 8316 winter wheat genotypes using the BluVision Macro (Macrobot) technique to identify MTAs associated with resistance to the same *Bgt* isolate.^40^ None of the 51 resistance loci were common with the resistance loci in our recent study. This could be due to the different types of marker data used in this study compared with ours, as Hinterberger *et al*. (2022)^40^ used GBS‐SNPs, which only weakly overlap with our markers. Furthermore, in our study, we used 197 spring wheat genotypes that could be different in their genome than the winter genotypes used in Hinterberger *et al*. (2022).^40^ Another key difference lies in the phenotyping methods employed. While the Macrobot focuses on scoring disease severity, which captures the final result of the entire infection process, the Microphenomics approach used in our study targets the early stages of plant‐pathogen interactions. This allows for a finer dissection of the infection process, enabling the discovery of novel resistance mechanisms that may not manifest in visible disease symptoms. For example, resistance mechanisms such as pathogen recognition, hypersensitive responses, or early halts in pathogen development can be missed when only the final infection outcome is assessed. Therefore, Microphenomics provides an additional layer of understanding by highlighting early resistance responses that may not correlate directly with traditional measures of disease severity.

Furthermore, it was reported that FAL‐92315 isolate is avirulent to *Pm1*, *Pm2*, *Pm3a*, *Pm3c*, *Pm3d*, *Pm4a*, *Pm4b*, *Pm5*, *Pm6*, *Pm8*, and *Pm9*.^40^ Previous studies reported that these genes distributed among eight chromosomes of the 21‐wheat chromosomes as follows: 6B (*Pm1*), 5D (*Pm2*), 1A (*Pm3*), 2A (*Pm4*), 7B (*Pm5*), 2B (*Pm6*), 1B (*Pm8*), and 7A (*Pm9*).^40^, ^70^, ^71^, ^72^ Due to the distribution of significant markers among 20 wheat chromosomes in this recent study, we can conclude that the evaluated wheat panel provides new genetic sources for early resistance to WPM.

To gain comprehensive insight into the functions of the 106 identified gene models in controlling the early resistance of WPM, gene enrichment analysis was performed. Gene enrichment analysis has been recognized as an effective approach for elucidating the genetic regulation of resistance to various environmental and biological stresses in plants.^39^, ^73^ A different number of pathways was identified by gene enrichment analysis based on BP, CC, and MF in this recent study (Fig. 4). Most of the pathways identified were linked to BP (17 pathways) that worked together in one network. It was determined that this network was regulated by five distinct gene models (*TRAESCS1A02G251700*, *TRAESCS1B02G262600*, *TRAESCS1B02G380100*, *TRAESCS1D02G251500*, and *TRAESCS1D02G360600*). These gene models were functionally characterized as regulating the production of cation‐transporting ATPase and a Serine/threonine‐protein kinase. Some transporting cations were reported previously to regulate disease resistance in some crops, such as rice and wheat.^74^, ^75^ Serine/threonine‐protein kinase has previously been identified as a crucial component of the powdery mildew resistance gene *Pm21*.^76^ Moreover, these five genes were involved in regulating processes within the endoplasmic reticulum, as evidenced by their annotation in the CC category (Table S4). Previous studies indicated that the endoplasmic reticulum is a key in modulating plant responses to both biotic stresses and environmental conditions.^77^ Therefore, this analysis underscores the importance of the five genes identified here in resistance to WPM. Most of the BP pathways were associated with pollen maturation and germination. Previous studies identified a correlation between pollen maturation and disease resistance in wheat, explaining the distorted inheritance of the stem rust resistance gene by the presence of a gene that induces pollen death.^78^

The five identified gene models, as well as the roles they play in controlling wheat's response to both biological and environmental stresses, are crucial for accelerating the future of wheat breeding for many significant stresses. The five significant markers located within these gene models could be converted into KASP markers to be tested in other genetic backgrounds. Indeed, further studies are required to better understand their role in improving early resistance to WPM, particularly their involvement in pollen maturation and wheat yield improvement. However, the inclusion of these genes in future breeding programs will enhance the level of WPM resistance, especially at the early stage of infection, thus preventing the progress of this harmful pathogen on wheat seedlings’ leaves.

### 4.3 Selection of elite genotypes with early resistance to WPM

In this recent study, the evaluation was conducted using a specific *Bgt* isolate (FAL 92315). This isolate has been used to evaluate different winter wheat genotypes and their seedling resistance for WPM.^40^ In our study, we indentified eight spring wheat genotypes that exhibited high levels of early WPM resistance. The highest level of resistance was found for ‘Ritchie’ genotype from the UK (Table 4). Half of the genotypes presenting high levels of early WPM resistance were from Oman (4 genotypes), with ‘Mufesgha’ showing the highest level of resistance.

The highest number of targeted alleles for the significant markers was observed in ‘Ritchie’, however, this number was quite similar across all eight selected genotypes (Fig. 5). The selected genotypes were distributed among three different clusters. In each cluster, genotypes originating from the same country were grouped together. Furthermore, the highest genetic distance values were found between the Omani cultivar ‘Mufesgha’ and all the German genotypes, as well as between it and ‘Ritchie’. Studies have indicated that the most suitable parents for crossing in breeding programs are those with a large genetic distance.^79^ Therefore, the UK genotype as well as Omani genotypes are good sources to improve the early resistance against WPM in European wheat genotypes. Integrating phenotypic selection with comprehensive genetic analyses, including population structure, genetic distance, and GWAS findings, has been reported as an effective strategy for identifying optimal parents to enhance target traits.^28^, ^80^, ^81^

## 5 CONCLUSION

In conclusion, the Microphenomics approach provided multiple traits representing early resistance to wheat powdery mildew. Unlike traditional methods that rely on visual scoring of disease severity at later stages, Microphenomics focuses on early plant‐pathogen interactions, enabling the discovery of resistance mechanisms that may not be detectable through final infection outcomes. The tested wheat panel exhibited high variance for all the studied parameters except for the median area of colonies after 48 h of infection. Furthermore, the low significant correlations between the studied parameters emphasize the importance of including all of them in evaluation and association mapping studies. This highlights the potential of Microphenomics to dissect the infection process in finer detail and uncover novel aspects of resistance.

The large number of markers was significantly correlated with resistance, along with their distribution across 21 different chromosomes, suggesting the presence of a complex genetic system that controls the early interaction between wheat seedlings and *Bgt*. This comprehensive approach not only expands our understanding of early resistance mechanisms but also provides valuable insights for breeding programs aiming to breed wheat varieties with improved and long‐lasting resistance to powdery mildew. The five genes identified in this study, along with their associated markers, may be utilized in marker‐assisted selection to enhance early resistance to WPM. Moreover, the eight selected genotypes, especially those from the UK and Oman, represent valuable genetic sources to improve early WPM resistance in European wheat germplasm.

## CONFLICT OF INTEREST STATEMENT

The authors declare no conflict of interests.

## AUTHOR CONTRIBUTION

A. M. I. M. helped in designing the experiment, helped in evaluating the genotypes, performed the genetic and phenotyping analysis, discussed the results, and drafted the manuscript. H. I. helped in evaluating the genotypes and helped in designing the experiment. S. L. performed the image analysis and quantification. A. B. reviewed the manuscript and helped in discussing the results. D. D. designed the experiment, helped in discussing the results, and reviewed the manuscript.

## FUNDING

This work was financially partially supported by the Alexander von Humboldt Foundation and German Federal Ministry of Education and Research (BMBF) grants FKZ 031B1304A to D. D. and H. I., and FKZ 031B1300A to D. D. and S. L. Open Access funding enabled and organized by Projekt DEAL.

## CLINICAL TRIAL NUMBER

Not applicable.

## Supporting information


**Data S1.** Supporting Information.

## Data Availability

Sequencing data that support the findings of this study are available on request from the corresponding author as they are used in ongoing projects.
