# Supplementary figures and images for: Unlocking the genetic control of early seedling resistance to wheat powdery mildew through microphenomics
Source: Pest Manag Sci. 2025 Oct 28;82(2):1668–79. doi: 10.1002/ps.70312 (PMC12790651; doi:10.1002/ps.70312)

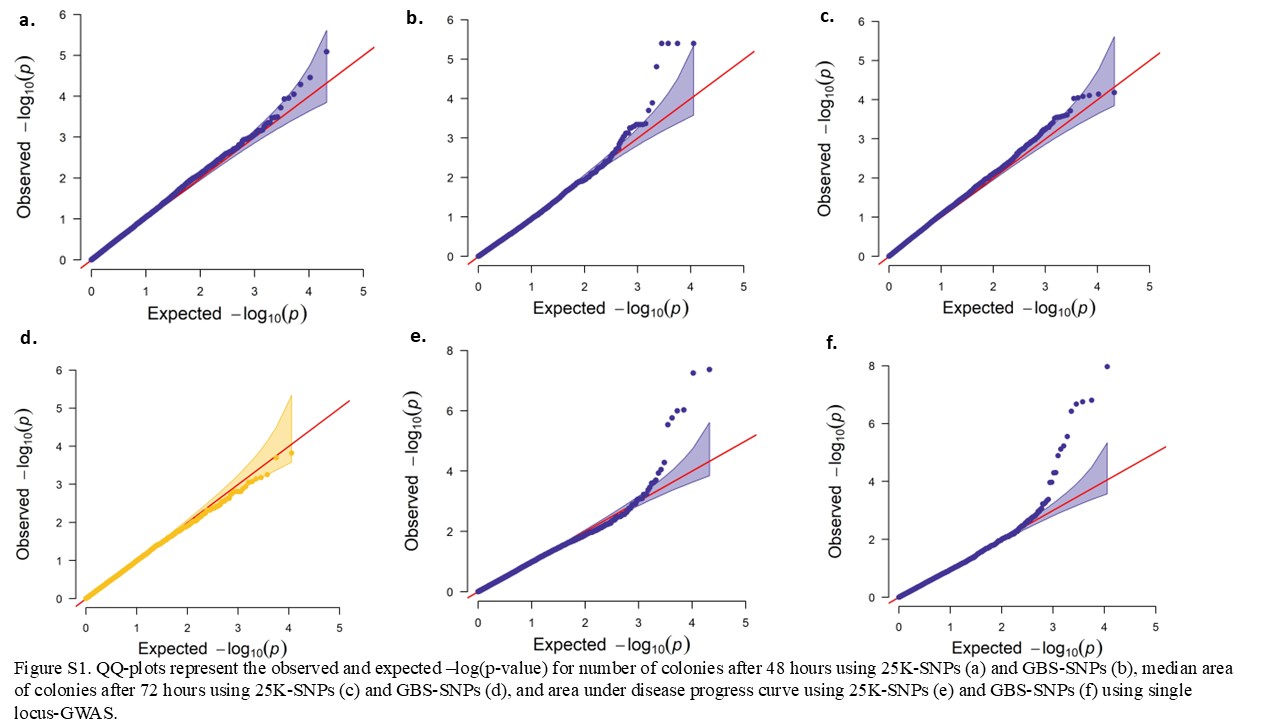

Supplement: Supplementary file 1 — Data S1. Supporting Information. [file PS-82-1668-s001.zip › ps70312-sup-0001-FigureS1@Figure S1.JPG]

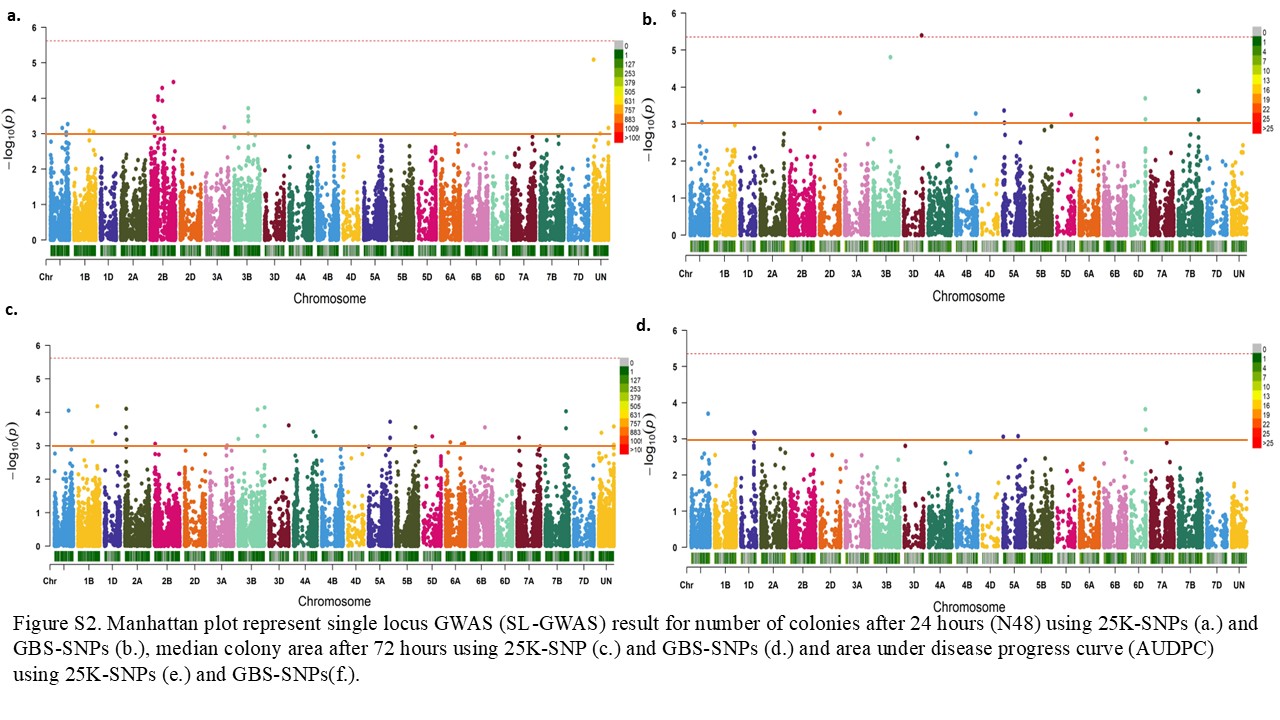

Supplement: Supplementary file 1 — Data S1. Supporting Information. [file PS-82-1668-s001.zip › ps70312-sup-0002-FigureS2@Figure S2.JPG]

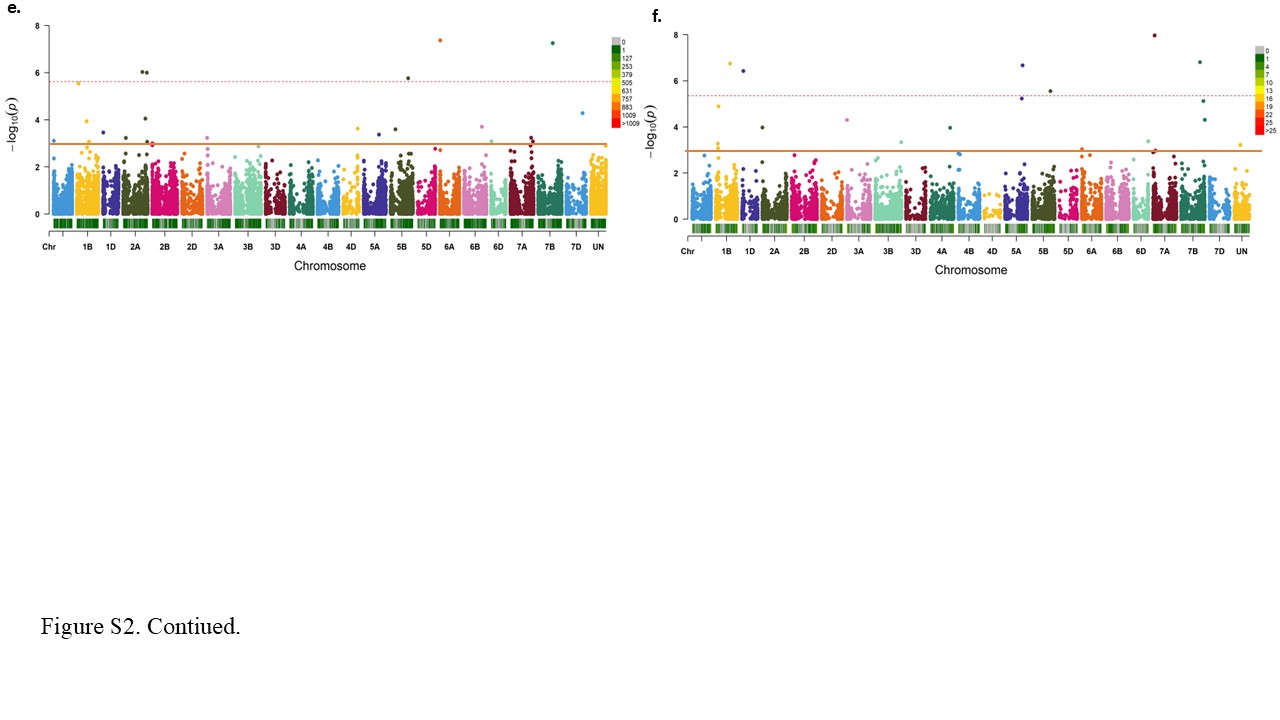

Supplement: Supplementary file 1 — Data S1. Supporting Information. [file PS-82-1668-s001.zip › ps70312-sup-0003-FigureS2@Figure S2_continued.JPG]

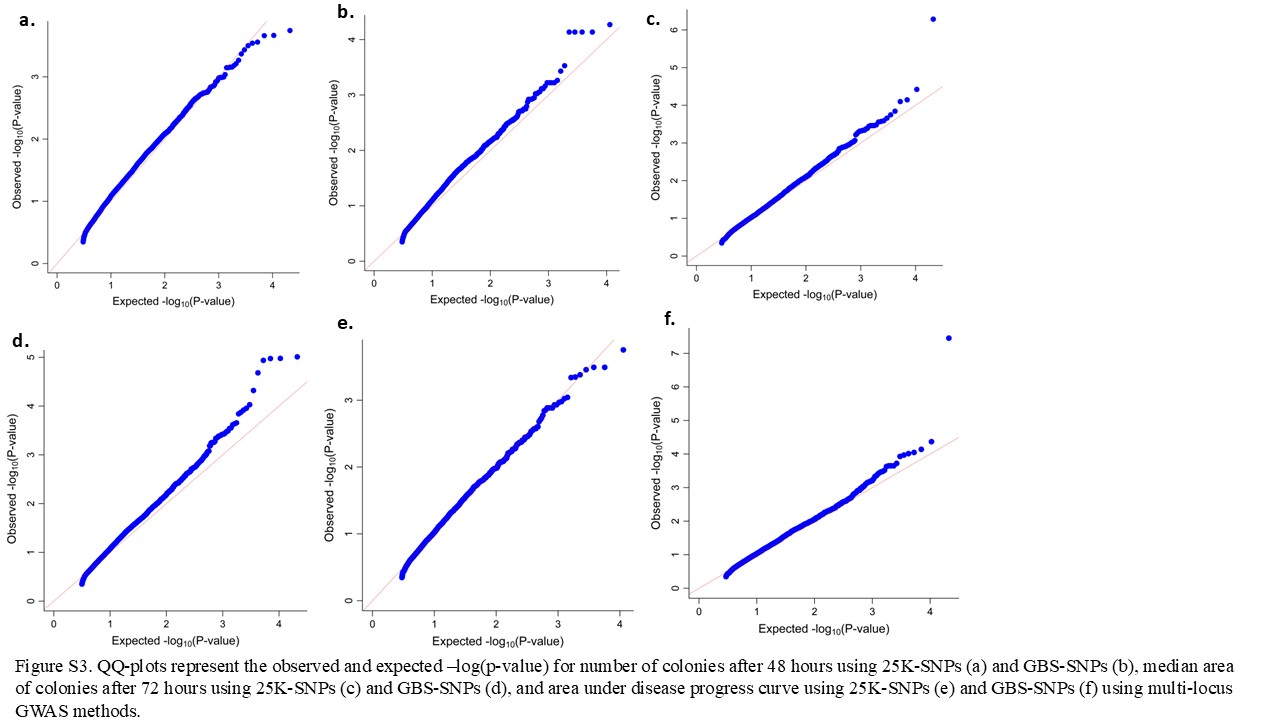

Supplement: Supplementary file 1 — Data S1. Supporting Information. [file PS-82-1668-s001.zip › ps70312-sup-0004-FigureS3@Figure S3.JPG]

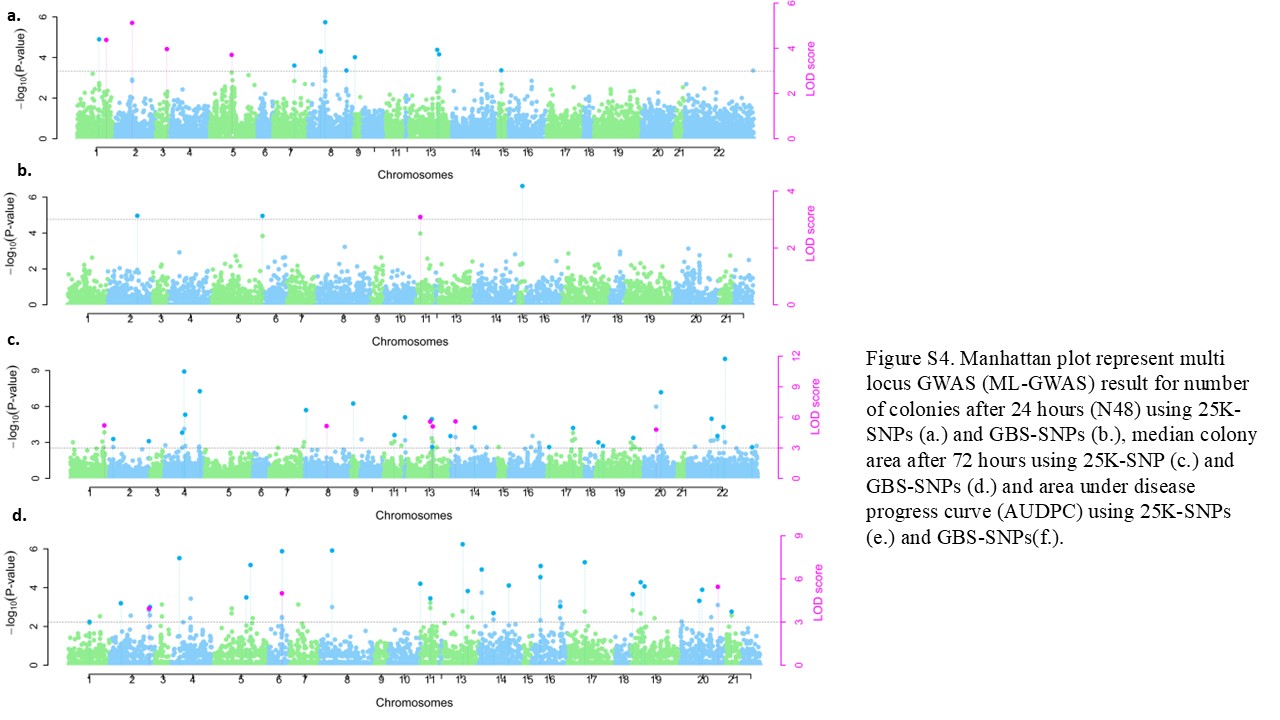

Supplement: Supplementary file 1 — Data S1. Supporting Information. [file PS-82-1668-s001.zip › ps70312-sup-0005-FigureS4@Figure S4.JPG]

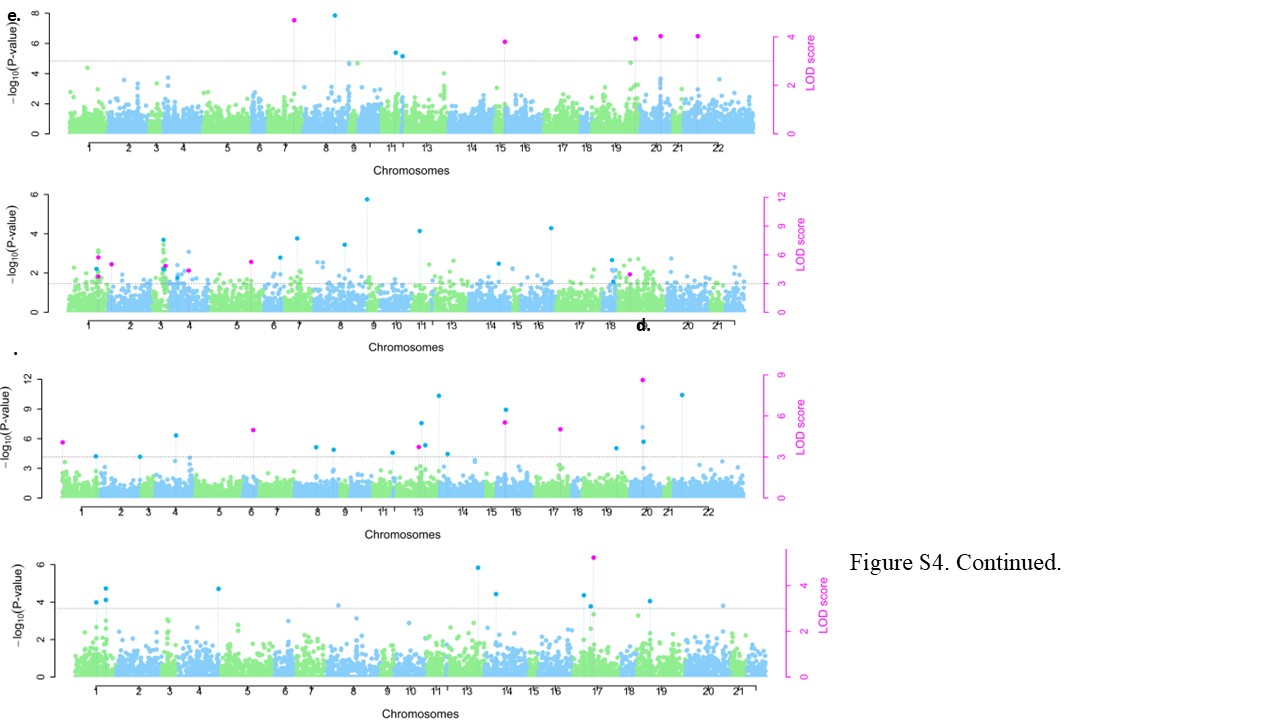

Supplement: Supplementary file 1 — Data S1. Supporting Information. [file PS-82-1668-s001.zip › ps70312-sup-0006-FigureS4@Figure S4_ continued.JPG]

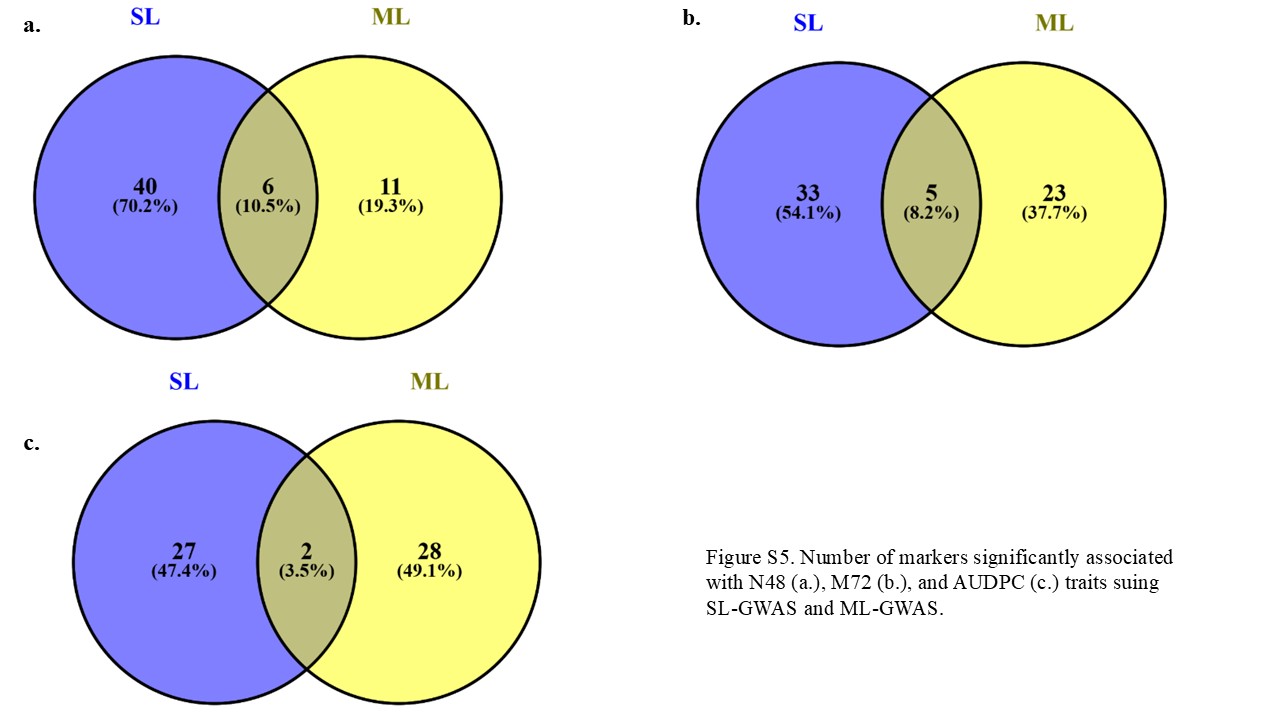

Supplement: Supplementary file 1 — Data S1. Supporting Information. [file PS-82-1668-s001.zip › ps70312-sup-0007-FigureS5@Figure S5.JPG]
